# Clinical and Radiographic Comparison of Oblique Lateral Lumbar Interbody Fusion and Minimally Invasive Transforaminal Lumbar Interbody Fusion in Patients with L4/5 grade‐1 Degenerative Spondylolisthesis

**DOI:** 10.1111/os.13360

**Published:** 2023-05-08

**Authors:** Da He, Wei He, Wei Tian, Bo Liu, Yajun Liu, Yuqing Sun, Yonggang Xing, Zhao Lang, Yumei Wang, Tengfei Ma, Mingming Liu

**Affiliations:** ^1^ Department of Spine Surgery Beijing Jishuitan Hospital Beijing China

**Keywords:** minimally invasive, oblique lumbar interbody fusion, percutaneous pedicle screw fixation, spondylolisthesis, transforaminal lumbar interbody fusion

## Abstract

**Objectives:**

To compare the clinical and radiographic outcomes of oblique lateral lumbar interbody fusion and minimally invasive transforaminal lumbar interbody fusion in patients with grade‐1 L4/5 degenerative spondylolisthesis.

**Methods:**

Based on the inclusion and exclusion criteria, the comparative analysis included consecutive patients with grade‐1 degenerative spondylolisthesis who underwent oblique LIF (OLIF, *n* = 36) or minimally invasive transforaminal LIF (MI‐TLIF, *n* = 45) at the Department of Spine Surgery, Beijing Jishuitan Hospital from January 2016 to August 2017. Patient satisfaction Japanese Orthopaedic Association score, visual analog scale (VAS) scores for back and leg pain, Oswestry disability index (ODI), radiographic outcomes including anterior/posterior disc heights (ADH/PDH), foraminal height (FH), foraminal width (FW), cage subsidence, cage retropulsion, and fusion rate were assessed during a 2‐year follow‐up. Continuous data are presented as mean ± standard deviation and were compared between groups using the independent sample t‐test. Categorical data are presented as *n* (%) and were compared between groups using the Pearson chi‐squared test or Fisher's exact test. Repetitive measurement and analysis of variance was employed in the analysis of ODI, back pain VAS score, and leg pain VAS score. Statistical significance was defined as *p* < 0.05.

**Results:**

The OLIF and MI‐TLIF groups comprised 36 patients (age, 52.1 ± 7.2 years; 27 women) and 45 patients (age, 48.4 ± 14.4 years; 24 women), respectively. Satisfaction rates at 2 years post procedure exceeded 90% in both groups. The OLIF group had less intraoperative blood loss (140 ± 36 *vs* 233 ± 62 mL), lower back pain VAS score (2.42 ± 0.81 *vs* 3.38 ± 0.47), and ODI score (20.47 ± 2.53 *vs* 27.31 ± 3.71) at 3 months follow‐up (with trends toward lower values at 2 years follow‐up), but higher leg pain VAS scores at all postoperative time points than the MI‐TLIF group (all *p* < 0.001). ADH, PDH, FD, and FW improved in both groups post‐surgery. At the 2 year follow‐up, the OLIF group had a higher rate of Bridwell grade‐I fusion (100% *vs* 88.9%, *p* = 0.046) and lower incidences of cage subsidence (8.33% *vs* 46.67%, *p* < 0.001) and retropulsion (0% *vs* 6.67%, *p* = 0.046) than the MI‐TLIF group.

**Conclusions:**

In patients with grade‐I spondylolisthesis, OLIF was associated with lower blood loss and greater improvements in VAS for back pain and ODI and radiologic outcomes than MI‐TLIF. The OLIF is more suitable for these patients with low back pain as the main symptoms are accompanied by mild or no leg symptoms before operation.

## Graphical abstract

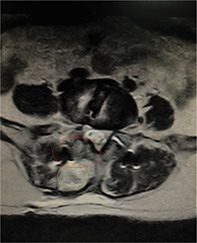

## Introduction

Symptoms of degenerative spondylolisthesis include back pain, leg pain, and neurogenic claudication^1^. Conventional open procedures such as anterior lumbar interbody fusion (ALIF), posterior lumbar interbody fusion (PLIF), and transforaminal lumbar interbody fusion (TLIF) have been applied with successful outcomes, although each technique has its advantages and disadvantages.

Emerging minimally invasive techniques, such as minimally invasive TLIF (MI‐TLIF) and oblique lumbar interbody fusion (OLIF), are progressively changing the treatment modalities for patients suffering from degenerative lumbar diseases. MI‐TLIF achieves direct decompression of spinal neural elements and was first described (using a tubular retractor) by Foley *et al*. in 2003^2^. OLIF was first introduced in 2012^3^, with the primary surgical goal being to implant the largest possible interbody cage into the area of surgical exposure to facilitate fusion rates, preserve posterior column structure, reduce paraspinal muscle trauma, maximize segmental lordosis, and correct sagittal imbalance^4^.

MI‐TLIF is a technically challenging operation often performed in a limited working space. A previous study utilizing MI‐TLIF reported that the total complication rate was 8.11%^5^, which was lower than previously described rates of 30.77%^6^ and 10.47%^7^. A meta‐analysis of 5454 patients published in 2015 concluded that the complication rate for MI‐TLIF was 19.2% overall, of which 2.2% were for neurologic deficits and 3.6% for intraoperative complications^8^.

OLIF utilizes the ante‐psoas muscle approach to exert an alternative mechanism of action, providing indirect neural decompression by expansion of the bony neuroforamen and distraction of the ligamentous stenosis of the central canal. In experiments on cadavers, Davis *et al*. investigated the oblique lateral corridor between the peritoneum and psoas muscle without dissecting or traversing the psoas muscle, and concluded that the corridor provides safe and easy access to the L2–S1 intervertebral discs with minimal psoas retraction^9^. Many clinicians consider OLIF to be relatively safe and a meta‐analysis study of 1453 patients published in 2017 demonstrated that the overall complication rate associated with OLIF was 11.4%, with the incidences of neurologic deficits and intraoperative complications being 4.2% and 1.5%, respectively^10^.

In this study, we performed a retrospective analysis to compare the clinical and radiographic outcomes of oblique lateral lumbar interbody fusion and minimally invasive transforaminal lumbar interbody fusion in patients with grade‐1 degenerative spondylolisthesis (L4/5). Our research questions included the following. First, are OLIF procedures performed on patients with grade‐1 degenerative spondylolisthesis (L4/5) feasible and safe? Second, does minimally invasive transforaminal lumbar interbody fusion result in a stable internal fixation, reduced surgical trauma, and early postoperative recovery? Third, what perioperative complications are associated with the two surgical techniques?

## Methods

### Study Design and Participants

**Fig. 1:**
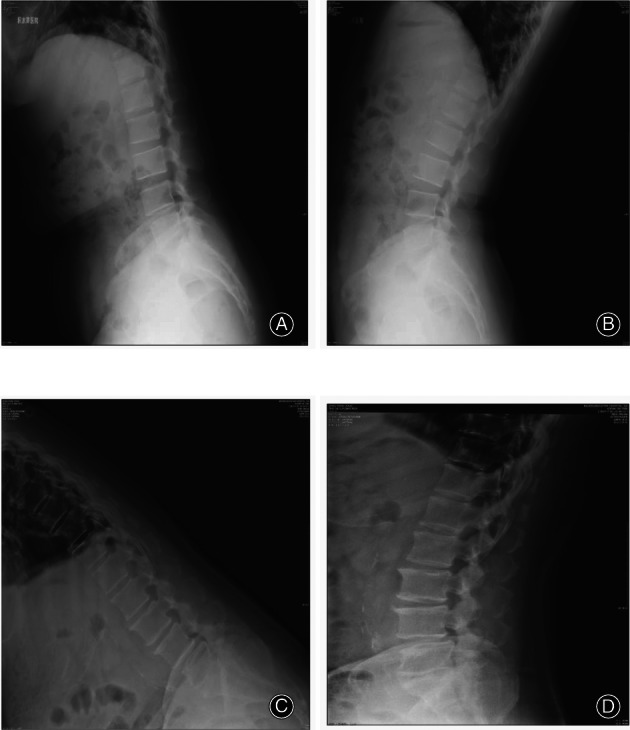
(A) Flexion radiograph of case 1 (MI‐TLIF). (B) Extension radiograph of case 1 (MI‐TLIF). It can be seen that the L4/5 joint gap becomes larger. (C) Flexion radiograph of case two (OLIF). (D) Extension radiograph of case 2 (OLIF). Spondylolisthesis of Meyerding grade 1 (slippage ≤25%) at the L4–L5 level was confirmed by radiography

This study was a retrospective analysis of patients with spondylolisthesis who underwent one of the two surgical procedures at our hospital between January 2016 and August 2017. The inclusion criteria were: (i) spondylolisthesis of Meyerding grade 1 (slippage ≤25%) at the L4–L5 level was confirmed by radiography (Figure 1); (ii) surgery was indicated due to symptoms of mechanical lower back pain that had not been relieved after more than 6 months of conservative treatment; (iii) because of the lumbar instability, patients with grade‐1 degenerative spondylolisthesis suffered from leg pain; (iv) follow‐up data were available for a minimum of 24 months. The exclusion criteria were: (i) spinal canal stenosis (central stenosis and foraminal stenosis); (ii) cauda equina syndrome; (iii) spinal tumor; (iv) spinal infection; (v) spinal fracture; and (vi) previous surgery at the L4–L5 level.

### Selection Criteria and Contraindications to OLIF

Criteria to OLIF: Foramina stenosis was caused by the decrease of the height of the foramina, which was caused by the decrease of the height of the intervertebral space.

Contraindications to OLIF: Foramina stenosis was mainly caused by hyperplasia.

For the analysis, the patients were divided into the OLIF group or the MI‐TLIF group based on the surgical technique used. The surgical method had been selected by the patient after they had been provided with information about the potential advantages and disadvantages of each approach.

This study was approved by the ethics committee of Beijing Jishuitan hospital (reference number: 201811‐03), and informed consent was waived due to the retrospective design of the study.

### Surgical Techniques

#### OLIF

OLIF and MI‐TLIF were performed under general anesthesia. The same chief surgeon, who had 24 years of experience in spinal surgery, performed all the operations. All patients were discharged 7 days after surgery.

OLIF was performed using a standard procedure^10^. A 4‐cm skin incision was made 6–10 cm anterior to the mid‐portion of the marked disc. The retroperitoneal space was accessed *via* blunt dissection, and the peritoneum was mobilized anteriorly to expose the anatomical oblique lateral corridor. An intervertebral cage (12 mm high, 50 mm long, and 18 mm wide; 6° lordotic; 3.27 ml graft volume; Clydesdale Spinal System, Medtronic, Memphis, TN, USA) filled with demineralized bone matrix (DBM; Wright Medical Technology Inc., Arlington, TN, USA) was inserted.

Subsequently, the patient was placed in the prone position to undergo posterior bilateral percutaneous pedicle screw fixation (CD Horizon Solera Voyager Spinal System, Medtronic). None of the patients in the OLIF group underwent additional laminectomy for the level of spondylolisthesis observed.

#### MI‐TLIF

MI‐TLIF was carried out using a standard method^11^. Unilateral MI‐TLIF was performed with the aid of a microscope, and a straight cage (12 mm high, 26 mm long, and 10 mm wide; 0° lordotic; 0.90 ml graft volume; Capstone Peek Spinal System, Medtronic) filled with demineralized bone matrix (DBM; Wright Medical Technology Inc., Arlington, TN, USA) was inserted.

Posterior bilateral percutaneous pedicle screw fixation (CD Horizon Solera Voyager Spinal System, Medtronic) was performed in all patients.

### Demographic and Operative Data

The following demographic and operative data were extracted from the medical records: sex, age, weight, BMI, and intraoperative blood loss.

### Follow‐up and Outcome Measures

The following assessments were made before surgery and postoperatively at 1 week, 3 months, and 2 years, respectively: visual analog scale (VAS) scores for back pain and leg pain; Oswestry Disability Index (ODI, version 2.0)^12^; anteroposterior (AP)/lateral radiography and flexion‐extension radiography; computed tomography (CT); and magnetic resonance imaging (MRI).

Patient satisfaction with treatment was determined using the Japanese Orthopaedic Association (JOA) scoring system^13^. Patient satisfaction with the clinical effect (with focus on the symptoms in the back and lower limbs) was graded as satisfactory, acceptable, or very unsatisfactory.

#### Japanese Orthopaedic Association (JOA)

The JOA score is composed of four sections: subjective symptoms (low back pain, leg pain, and gait), clinical signs (straight‐leg‐raising test, sensory and motor disturbances), restriction of activities of daily living (seven items), and urinary bladder function as minus points.

#### Oswestry Disability Index (ODI)

Oswestry Disability Index (ODI) is a principal, condition‐specific outcome measure used in the management of spinal disorders, and to assess patient progress in routine clinical practice. A value of 0%–20% is considered mild dysfunction, 21%–40% is moderate dysfunction, 41%–60% is severe dysfunction, and 61%–80% is considered as disability. For cases with score of 81%–100%, they are considered as either long‐term bedridden, or exaggerating the impact of pain on their life.

#### Lower Back and Leg Pain

The visual analog scale (VAS) of the lower limbs and back were analyzed to evaluate the effects of treatment on lower limb symptoms. Using a VAS ruler, A higher score indicated greater pain intensity. Patients described their lower leg pain intensity as 0 (no pain) to 10 (worst pain ever).

#### Cage Position

Evaluation of cage position was based on the imaging investigations. Cage subsidence was defined as being present if a cage was observed to sink into an adjacent vertebral body by >2 mm, based on comparisons with previous CT images^14^. Cage migration was defined as posterior movement of the cage by ≥3 mm compared with the immediate postoperative state.

#### Disc Measurements

**Fig. 2:**
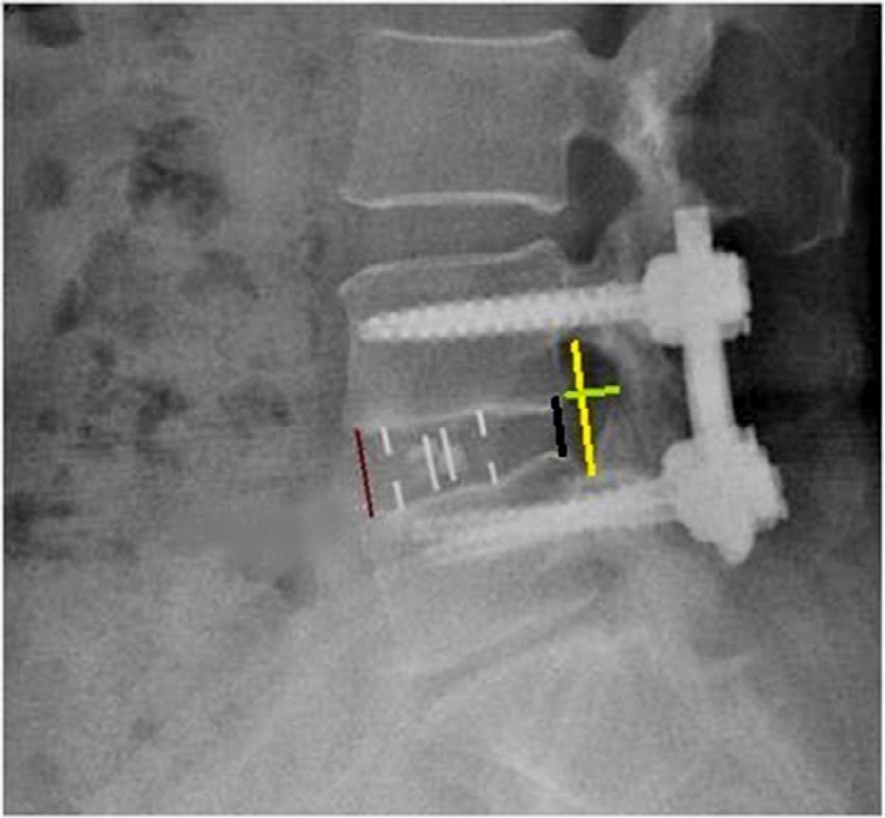
Radiological measurements used in this study. Lateral X‐ray image of lumbar vertebrae. There was OLIF Cage in the L4/5. The yellow line shows the foraminal height (FH). The red line shows the anterior disc height (ADH). The black line shows the posterior disc height (PDH), The green line shows the foraminal width (FW)

**Fig. 3:**
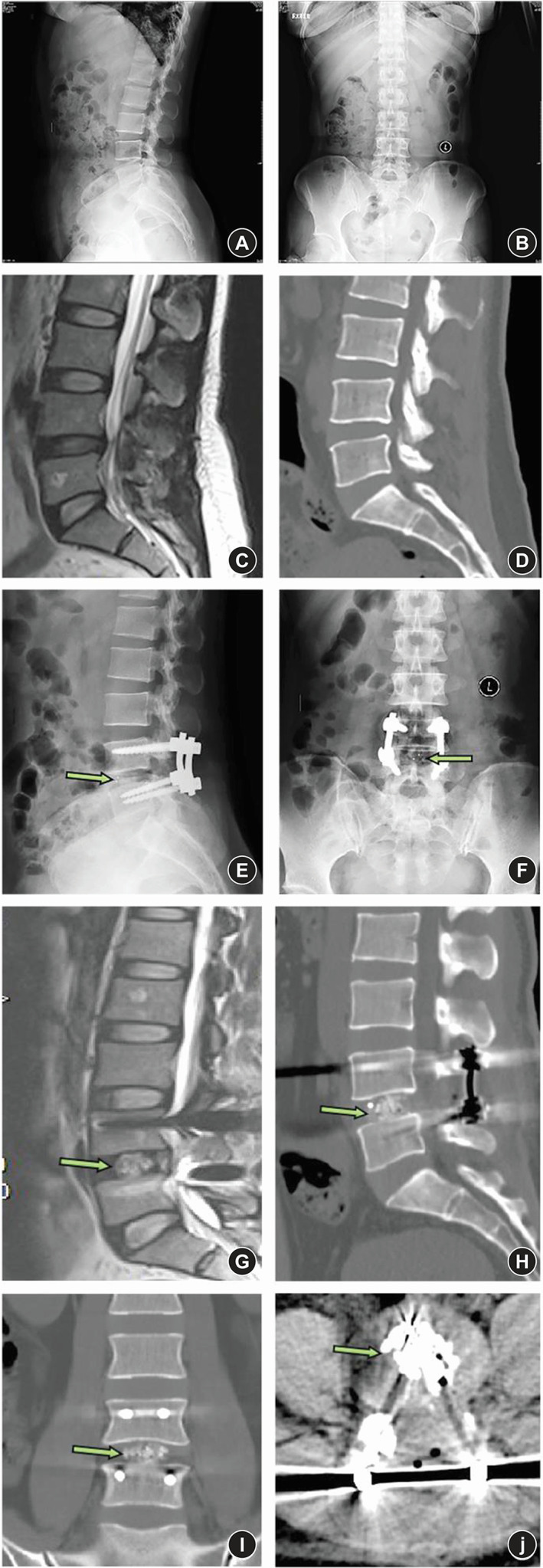
Case 1 (MI‐TLIF). (A) Sagittal X ray image before MI‐TLIF. (B) Anteroposterior X ray image before MI‐TLIF. (C) Sagittal MR image before MI‐TLIF. (D) Sagittal CT image before MI‐TLIF. (E) Sagittal X ray image after MI‐TLIF. The green arrow shows the marker of the cage. (F) Anteroposterior X ray image after MI‐TLIF. The green arrow shows the marker of the cage. (G) Sagittal MR image after MI‐TLIF. The green arrow shows the cage. (H) Sagittal CT image after MI‐TLIF. The green arrow shows the cage. (I) Coronal position CT image after MI‐TLIF. The green arrow shows the cage. (J) Cross section CT image after MI‐TLIF. The green arrow shows the cage

**Fig. 4:**
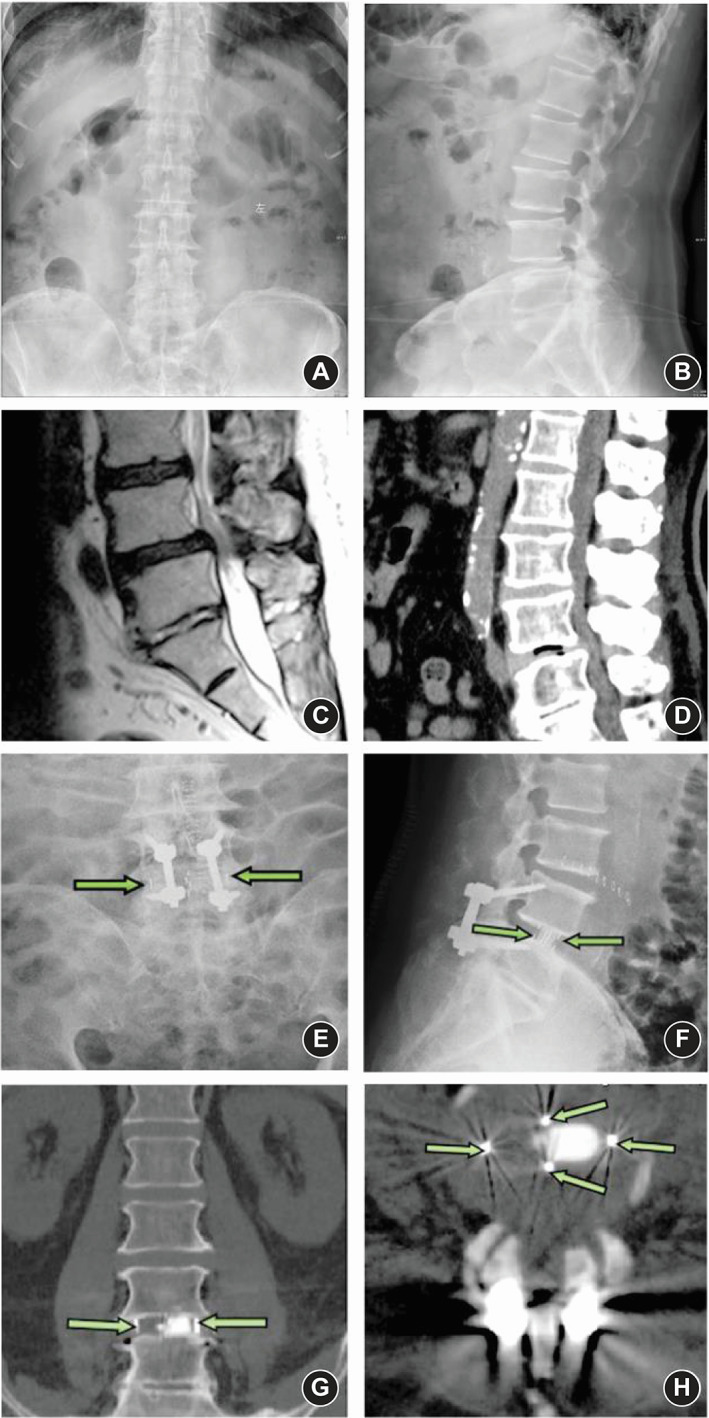
Case 2 (OLIF). (A) Anteroposterior X ray image before OLIF. (B) Sagittal X ray image before OLIF. (C) Sagittal MR image before OLIF. (D) Sagittal CT image before OLIF. (E) Anteroposterior X ray image after OLIF. The green arrows show the left marker and right marker of the cage. (F) Sagittal X ray image after OLIF. The green arrows show the front and back markers of the cage. (G) Cross‐section CT image after OLIF. The green arrows show the left and right markers of the cage. (H) Coronal position CT image after OLIF. The four green arrows show the four markers around the cage

Radiologic measurements (Figure 2) included: (1) Disc height (DH) (including anterior disc height [ADH] and posterior disc height [PDH]), (2) foraminal height (FH), (3) foraminal width (FW), and (4) fusion rate. ADH/PDH was defined as the distance of the anterior/posterior position from the upper to the lower endplate of the L4–5 level. FH was defined as the distance from the lower position of the pedicle of L4 to the upper position of the pedicle of L5. FW was defined as the distance from the lower posterior horn of the vertebral body of L4 to the vertex of the superior joint of L5. Fusion grading criteria were based on the Bridwell interbody fusion grading system^15^. Fusion rate was evaluated postoperatively at 3 months and 2 years, respectively, through the CT images. Use MI‐TLIF or OLIF surgery to list the preoperative and postoperative MR, CT, and X‐ray images of a patient respectively (Figures 3 and 4).

### Statistical Analysis

Statistical analyses were performed using SPSS 18.0 (SPSS Inc., Chicago, IL, USA). Continuous data are presented as mean ± standard deviation and were compared between groups using the independent sample t‐test. Categorical data are presented as *n* (%) and were compared between groups using the Pearson chi‐squared test or Fisher's exact test. Repetitive measurement and analysis of variance was employed in the analysis of ODI, back pain VAS score, and leg pain VAS score. Statistical significance was defined as *p* < 0.05.

## Results

### Demographic Profile and Operative Characteristics of Patients

A total of 81 patients were included in the analysis, and demographic profile and operative characteristics of patients are presented in Table 1. There was significantly less intraoperative blood loss in the OLIF group than in the MI‐TLIF group (140 ± 36 ml *vs* 233 ± 62 ml, *p* < 0.001).

**TABLE 1:** Demographic profile and operative characteristics of patients in the two groups

Characteristic	OLIF group (*n* = 36)	MI‐TLIF group (*n* = 45)	*p*
Sex			0.043^a^
Male	9 (25.0%)	21 (46.7%)	
Female	27 (75.0%)	24 (53.3%)	
Weight (kg)	65 ± 3.1	71 ± 2.5	0.077^b^
BMI (kg/m^2^)	23 ± 1.9	21 ± 2.5	0.062^b^
Age (years)	52.1 ± 7.2	48.4 ± 14.4	0.097^b^
Intraoperative blood loss (mL)	140 ± 36	233 ± 62	<0.001^b^

*Note*: Data are presented as *n* (%) or mean ± standard deviation. MI‐TLIF: minimally invasive transforaminal lumbar interbody fusion; OLIF: oblique lumbar interbody fusion.

^a^ chi‐squared test.

^b^ t‐test.

### Follow‐up Time

At 24 months (OLIF group 24 ± 1.7 months *vs* MI‐TLIF group 23 ± 2.2 months), 76/81 of patients (93.8%) returned to our hospital for their follow‐up visit. Follow‐up was conducted by telephone for the five patients who lived too far from our hospital to return at 24 months, and their 24‐month CT and MRI scans (obtained at a more local institution) were sent to our hospital for analysis. Thus, follow‐up data were available for all 81 patients.

### Clinical Outcomes

**Fig. 5:**
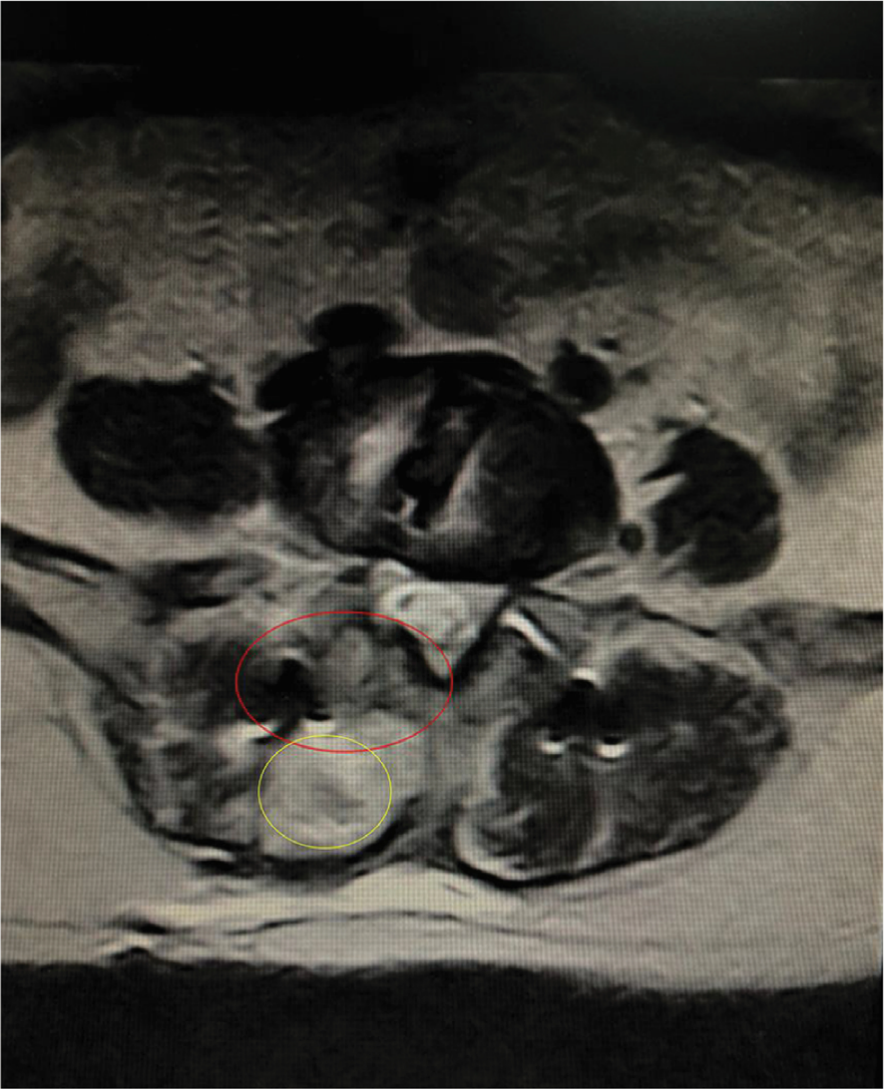
Tissue injury resulting from minimally invasive transforaminal lumbar interbody fusion (MI‐TLIF). The red circle highlights muscular tissue injury near the spinous process (2 years after surgery) caused by the surgical approach during MI‐TLIF

Patient satisfaction rate 2 years post‐surgery (Table 2) was more than 90% in both groups and was not significantly different between the OLIF group (91.7%) and MI‐TLIF group (91.1%). Preoperatively, there were no significant differences between the OLIF and MI‐TLIF groups in back pain VAS score or ODI score (Table 2). Both groups showed progressive improvements in all clinical outcome scores during postoperative follow‐up, as compared with preoperative values. Back pain VAS scores were significantly lower in the OLIF group than in the MI‐TLIF group at 3 months after surgery (2.42 ± 0.81 *vs* 3.38 ± 0.47, *p* < 0.001). Milder back pain symptoms in the OLIF group at 3 months may have been due to less iatrogenic violation of the posterior lumbar elements than that which occurs after MI‐TLIF (Figure 5). There was also a trend toward a lower value in the OLIF group at 2 years postoperatively (0.86 ± 0.64 *vs* 1.07 ± 0.25, *p* = 0.052; Table 2). ODI score was also lower in the OLIF group than in the MI‐TLIF group at 1 week (33.61 ± 2.10 *vs*. 34.75 ± 2.16, *p* = 0.019) and 3 months (20.47 ± 2.53 *vs* 27.31 ± 3.71, *p* < 0.001), with a trend toward a lower value at 2 years postoperatively (14.89 ± 1.93 *vs* 15.82 ± 1.45, *p* = 0.074; Table 2).

**TABLE 2:** Comparison of clinical outcome scores between the two groups

Measure	OLIF group (*n* = 36)	MI‐TLIF group (*n* = 45)	*p*
Back pain VAS score			
Preoperative	8.4 ± 0.8	8.2 ± 0.3	0.510^b^
Postoperative, 1 week	5.6 ± 0.8	5.4 ± 0.7	0.280^b^
Postoperative, 3 months	2.4 ± 0.8	3.4 ± 0.5	0.000^b^
Postoperative, 2 years	0.9 ± 0.6	1.1 ± 0.3	0.052^b^
Leg pain VAS score			
Preoperative	5.6 ± 1.0	7.7 ± 0.9	<0.001^b^
Postoperative, 1 week	3.2 ± 0.8	2.0 ± 0.6	<0.001^b^
Postoperative, 3 months	2.9 ± 0.9	2.0 ± 0.7	<0.001^b^
Postoperative, 2 years	2.3 ± 0.6	1.4 ± 0.5	<0.001^b^
Oswestry disability index score			
Preoperative	44.7 ± 3.4	45.3 ± 1.6	0.312^b^
Postoperative, 1 week	33.6 ± 2.1	34.8 ± 2.1	0.019^b^
Postoperative, 3 months	20.5 ± 2.5	27.3 ± 3.7	<0.001^b^
Postoperative, 2 years	14.9 ± 1.9	15.8 ± 1.5	0.074^b^
Patient satisfaction at 2 years			1.000^a^
Satisfied	33 (91.7%)	41 (91.1%)	
Not satisfied	3 (8.3%)	4 (8.9%)	

*Note*: Data are presented as *n* (%) or mean ± standard deviation. MI‐TLIF, minimally invasive transforaminal lumbar interbody fusion; OLIF, oblique lumbar interbody fusion; VAS, visual analog scale. Patient satisfaction was determined using the Japanese Orthopaedic Association scoring system.

^a^ chi‐squared test.

^b^ t‐test.

### Radiologic Outcomes

ADH was significantly higher in the OLIF group than in the MI‐TLIF group at all postoperative time points (*p* = 0.015 at 1 week, *p* = 0.014 at 3 months and *p* = 0.024 at 2 years) despite being significantly lower preoperatively (*p* < 0.001; Table 3).

**TABLE 3:** Comparison of radiologic outcomes between the two groups

Measure	OLIF group (*n* = 36)	MI‐TLIF group (*n* = 45)	*p*
Anterior disc height (mm)			
Preoperative	4.79 ± 1.6	7.26 ± 2.0	<0.001^b^
Postoperative, 1 week	12.82 ± 1.1	12.15 ± 0.9	0.015^b^
Postoperative, 3 months	12.78 ± 1.6	12.08 ± 0.8	0.014^b^
Postoperative, 2 years	12.44 ± 1.1	12.00 ± 0.9	0.024^b^
Posterior disc height(mm)			
Preoperative	4.59 ± 2.0	6.07 ± 1.8	0.001^b^
Postoperative, 1 week	8.53 ± 1.7	11.32 ± 0.9	<0.001^b^
Postoperative, 3 months	8.32 ± 1.6	9.23 ± 0.6	0.003^b^
Postoperative, 2 years	8.07 ± 1.6	8.12 ± 0.6	0.868^b^
Foraminal height (mm)			
Preoperative	12.37 ± 3.6	13.13 ± 2.9	0.278^b^
Postoperative, 1 week	16.27 ± 4.0	17.01 ± 2.3	0.322^b^
Postoperative, 3 months	16.21 ± 3.8	15.96 ± 3.0	0.702^b^
Postoperative, 2 years	16.11 ± 3.8	14.55 ± 2.3	0.132^b^
Foraminal width (mm)			
Preoperative	7.28 ± 2.2	8.75 ± 1.2	0.002^b^
Postoperative, 1 week	9.47 ± 1.9	10.81 ± 1.2	0.003^b^
Postoperative, 3 months	9.35 ± 1.8	10.71 ± 2.2	0.002^b^
Postoperative, 2 years	9.21 ± 1.9	10.59 ± 1.2	0.004^b^
Bridwell interbody fusion			
Postoperative, 3 months			0.042^a^
Grade I	30 (83.3%)	28 (62.2%)	
Grade II	4 (11.1%)	10 (22.2%)	
Grade III	2 (5.6%)	7 (15.6%)	
Grade IV	0	0	
Postoperative, 2 years			0.046^a^
Grade I	36 (100%)	40 (88.9%)	
Grade II	0	2 (4.4%)	
Grade III	0	0	
Grade IV	0	3 (6.7%)	

*Note*: Data are presented as *n* (%) or mean ± standard deviation. MI‐TLIF: minimally invasive transforaminal lumbar interbody fusion; OLIF: oblique lumbar interbody fusion.

^a^ chi‐squared test.

^b^ t‐test.

PDH was significantly higher in the MI‐TLIF group than in the OLIF group preoperatively (*p* = 0.001) and at 1 week (*p* < 0.001) and 3 months (*p* = 0.003) postoperatively (Table 3). However, PDH did not differ between groups at 2 years postoperatively (Table 3).

The main reason for the relief of clinical symptoms was the increase of foraminal height. Therefore, the baseline between the two groups was not anterior disc height (mm) or posterior disc height (mm), but foraminal height. There was no significant difference in the FH baseline (preoperative) between two groups.FH and FW showed improvements after surgery in both groups (Table 3). FH did not differ significantly between the OLIF and MI‐TLIF groups postoperatively (Table 3). The cage used for the OLIF group (50 mm long, 18 mm wide, and 12 mm in height) had a larger cross‐sectional area than that used for the MI‐TLIF group (26 mm long, 10 mm wide, and 12 mm in height). However, FW was significantly higher in the MI‐TLIF group than in the OLIF group preoperatively (*p* = 0.002) and at 1 week (*p* = 0.003), 3 months (*p* = 0.002), and 2 years (*p* = 0.004) postoperatively (Table 3).

The rate of complete fusion (grade I according to Bridwell's criteria) was significantly higher in the OLIF group than in the MI‐TLIF group at 3 months (83.3% *vs* 62.2%, *p* = 0.042) and at 24 months (100% *vs* 88.9%, *p* = 0.046).

### Cage Position

**Fig. 6:**
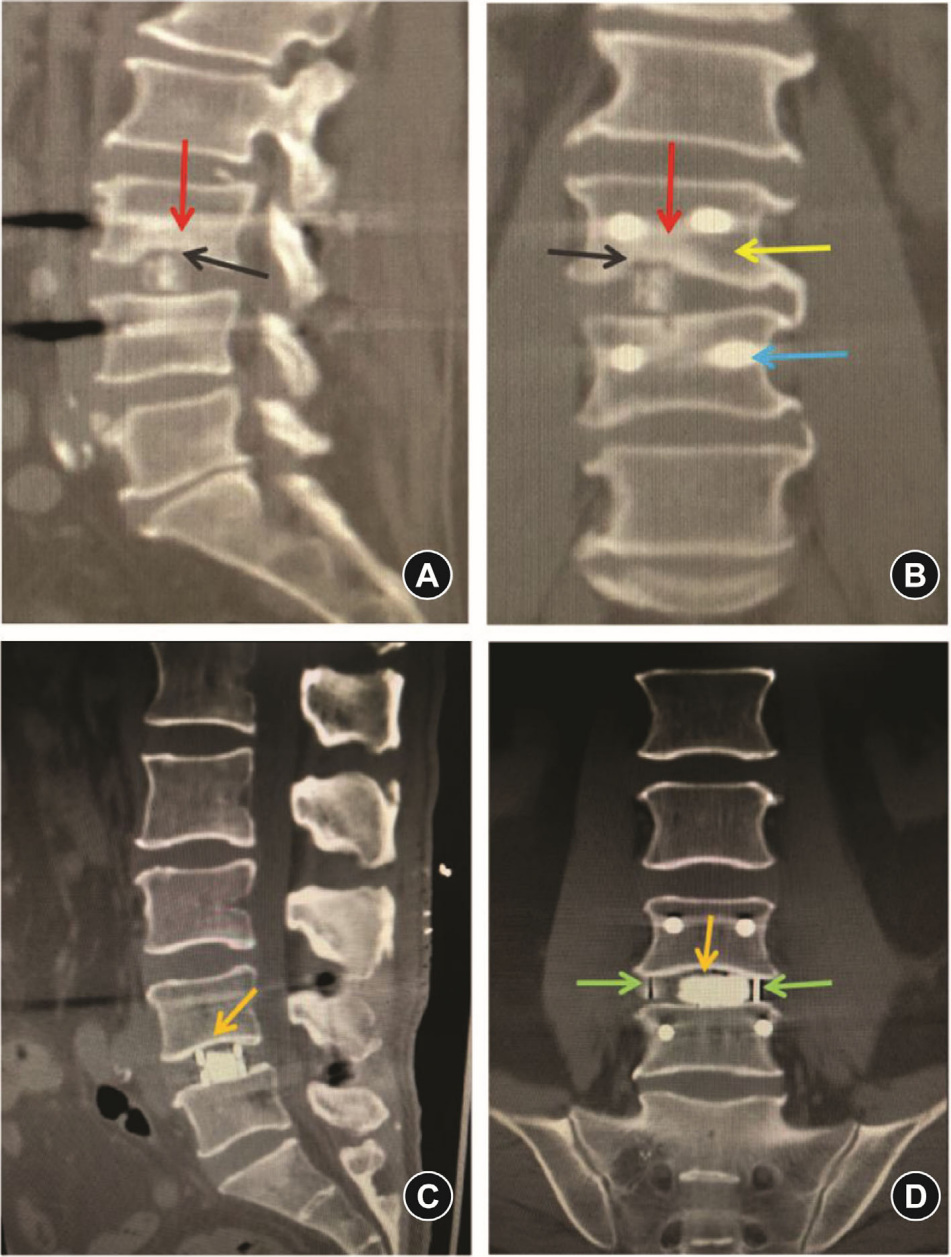
Computed tomography (CT) images after oblique lumbar interbody fusion (OLIF) and minimally invasive transforaminal lumbar interbody fusion (MI‐TLIF). (A) Sagittal CT image after MI‐TLIF. (A) Compression fracture occurred in the middle and lower part of the L3 vertebral body, which showed a marked increase in cancellous bone density in the vertebral body (red arrow). The lower end plate of L3 vertebral body collapsed (black arrow). The cage protrudes into the L3 vertebral body. (B) Anteroposterior image after MI‐TLIF. The cage was biased to the right of the vertebral body. There was a compression fracture in the middle and lower part of the right side of the L3 vertebral body, and the cancellous bone density on the right side of the vertebral body is significantly increased (red arrow). The bone density of the left vertebral body is normal (yellow arrow). The lower end plate of L3 vertebral body collapsed (black arrow). The cage protrudes into the L3 vertebral body. The cross section of the pedicle screw is shown by the blue arrow. (C) Sagittal CT image after OLIF. The lower end plate of L4 vertebral body and the upper end plate of L5 vertebral body were intact (orange arrows). There was no abnormal cancellous bone density in L4 and L5 vertebrae. (D) Anteroposterior image after OLIF. The markers on the left and right sides of the cage shown by green arrows, and the width of the cage is the same as the width of the vertebral body. The lower end plate of L4 vertebral body and the upper end plate of L5 vertebral body are intact (orange arrows). There was no abnormal cancellous bone density in L4 and L5 vertebrae

Cage subsidence (Figure 6) occurred more commonly in the MI‐TLIF group than in the OLIF group at 3 months postoperatively (35.6% *vs* 8.3%, *p* = 0.003) and at 24 months postoperatively (46.7% *vs* 8.3%, *p* < 0.001; Table 4). In addition, cage retropulsion did not occur in the OLIF group during the 2‐year follow‐up but was observed in three patients (6.7%) in the MI‐TLIF group (*p* = 0.046; Table 4).

**TABLE 4:** Complications

Complication	OLIF group (*n* = 36)	MI‐TLIF group (*n* = 45)	*p*
Intraoperative complications			
Segmental artery injury	3 (8.3%)	0	0.033
Dural tear	0	2 (4.4%)	0.105
Screw malposition	0	0	—
Early postoperative complications			
Leg weakness/numbness	4 (11.1%)	2 (4.4%)	0.255
Sympathetic chain injury	5 (13.9%)	0	0.004
Cerebrospinal fluid leak	0	2 (4.4%)	0.105
Infection	0	0	
Cage subsidence	3 (8.3%)	16 (35.6%)	0.003
Cage retropulsion	0	3 (6.7%)	0.046
Late postoperative complications			
Leg pain/numbness	0	3 (6.7%)	0.046
Adjacent segment disease	0	0	
Deep vein thrombosis	0	0	
Cage subsidence	3 (8.3%)	21 (46.7%)	<0.001
Cage retropulsion	0	3 (6.7%)	0.046

*Note*: Data are presented as *n* (%). MI‐TLIF, minimally invasive transforaminal lumbar interbody fusion; OLIF, oblique lumbar interbody fusion. *p*‐values were determined using the chi‐squared test.

### Complications

The intraoperative and postoperative complications are listed in Table 4. In the OLIF group, three cases of L4 segmental artery injury were noticed during surgery, and rapid hemostasis was achieved with a hemoclip. Lumbar sympathetic trunk injury is usually characterized by elevated skin temperature, reduced perspiration, paresthesia, skin discoloration, swelling of the lower limb on the surgical side. Five patients (13.9%) in the OLIF group had sympathetic injury and four patients (11.1%) had leg weakness/numbness which was transient in nature and they recovered within the first 3 months after surgery without the need for intervention (Table 4).

In the MI‐TLIF group, two patients (4.4%) suffered intraoperative dural tears during nerve decompression and developed cerebrospinal fluid leak. Three patients in the MI‐TLIF group complained of leg pain/numbness at 24 months after surgery. These three patients had cage retropulsion and a fusion grade of IV, and their pain resolved after revision surgery to reinsert the cage and compress the adjacent vertebrae. Two patients in the MI‐TLIF group suffered right L5 root palsy due to a local hematoma but recovered within 3 months.

## Discussion

The present study compared the effects of oblique lateral lumbar interbody fusion and minimally invasive transforaminal lumbar interbody fusion in patients with grade‐1 degenerative spondylolisthesis (L4/5).The findings showed that back pain VAS score at 3 months was significantly lower in the OLIF group than in the MI‐TLIF group. Milder back pain symptoms in the OLIF group at 3 months may have been due to less iatrogenic violation of the posterior lumbar elements than that which occurs after MI‐TLIF. An additional advantage of OLIF over MI‐TLIF is that the cage can achieve greater sagittal angle improvement by being implanted in the front third of the disc, which helps relieve tension in the paravertebral muscles. It was also notable that the ODI score at 3 months was significantly lower in the OLIF group than in the MI‐TLIF group, suggesting that OLIF may have advantages over MI‐TLIF with regard to achieving the intended surgical outcomes.

The incidence of cage subsidence as a complication of lateral lumbar interbody fusion (LLIF) was 10.84% in this study^14^. Three cases of endplate injury occurred in the OLIF group, and CT examination at the 2‐year follow‐up confirmed that three cages (8.3%) had shown subsidence. By contrast, the 2‐year follow‐up revealed cage subsidence in 21 patients (46.7%) in the MI‐TLIF group. Accompanying phenomena observed in the MI‐TLIF group were postoperative decreases in PDH and FH between 1 week and 2 years. One possible explanation for the above results is that the intervertebral space occupied by the cage was wider in the OLIF group (cross‐sectional cage area of 900 mm^2^) than in the MI‐TLIF group (cross‐sectional cage area of 260 mm^2^). The wider footprint of the cage used for OLIF may provide a more effective biological environment for the fusion process, reducing the chances of cage subsidence. Consistent with this proposal, Tohmeh *et al*. found that severe subsidence (≥4 mm) was more likely for a 50‐mm cage than for a 60‐mm cage^16^. A second possible reason is that the OLIF cage is implanted more securely on the dense ring apophysis as it runs through both sides of the endplate and is located anteriorly, in the strongest part of the endplate, whereas the MI‐TLIF cage is mostly located in the central, weaker part of the endplate. Xu *et al*. reported a significantly higher rate of endplate damage with the transforaminal approach (48%) than with the lateral approach (4%)^17^. Among 178 patients followed‐up for 25 months after LLIF, Malham *et al*. identified 13 patients (14 operative segments) with cage subsidence, with all cases occurring in the inferior endplates^18^. It is important that surgeons take care during preparation of the endplates to avoid endplate injury.

### Cage Retropulsion

Cage retropulsion may result in the loss of lumbar lordosis, a narrowing of the disc space and foramina, direct compression of the nerve roots and a lower fusion rate^19^, ^20^. In this study, we found a higher incidence of cage retropulsion in the MI‐TLIF group than in the OLIF group (no cases of cage retropulsion). We consider that the use of a lateral annular incision maintained the integrity of the posterior longitudinal ligament and posterior annulus in the OLIF group, which can theoretically prevent cage retropulsion. Buttermann *et al*. proposed that proper annular tension may reduce the risk of implant migration^21^. In addition, the sacral slope and pelvic incidence were greater in these three patients than in the other patients. Several studies have reported that interbody implants at L5–S1 are at greater risk of retropulsion^19^, ^22^.

### Complications

The most common complications of OLIF are lumbar sympathetic trunk injury and segmental artery injury, and neurogenic pain is commonly aggravated at night^23^. In this study, the probability of lumbar sympathetic trunk injury was higher for OLIF than for MI‐TLIF. In the OLIF group, there were five cases (13.9%) of anterolateral thigh pain or numbness, all of which were due to sympathetic chain injury, and three cases (8.3%) of segmental artery injury. Other authors have described similar findings. Hrabaleka *et al*. showed that the symptoms of sympathetic nerve injury can last up to 53 months^23^. Jin *et al*. reported that complications of OLIF occurred in three of 21 patients (leg paresthesia in two and local hematoma in one)^24^. One possible reason for the occurrence of lumbar sympathetic trunk injury during OLIF is that if the incision is not properly planned, excessive pressure on the psoas could result in injury to the genitofemoral nerve and sympathetic chain. Due to the specific anatomic path used for OLIF, the possibility of nerve root injury during surgery is lower. We suggest that measures to minimize nerve injury and reduce neurologic symptoms should include a clear visual field, careful performance of the operation, especially during separation of the psoas major and vascular sheath, and avoidance of electric coagulation/use of an electric knife.

### Limitations

This study had several limitations. First, this was a retrospective study, so the analysis may have been affected by information bias or selection bias. Second, this was a single‐center study with a small sample size. Third, the follow‐up time was limited to 2 years, so longer‐term outcomes were not evaluated. Fourth, some of the indicators should be undertaken on subgroup analysis based on age, gender, classification in the future research.

## Conclusions

In patients with grade‐I spondylolisthesis, OLIF was associated with less blood loss and better improvements in some clinical (VAS for back pain and ODI) and radiologic outcomes than MI‐TLIF, although patients in the OLIF group had higher leg pain VAS scores than patients in the MI‐TLIF group. As the greatest limitation of OLIF is indirect decompression, the ability to relieve symptoms of lower limbs is limited. Our advice is to choose patients with low back pain as the main symptoms, accompanied by mild or no leg symptoms before operation. Moreover, during OLIF surgery, particular attention should be paid to the lumbar sympathetic trunk to reduce postoperative neurologic complications.

## Competing Interests

All authors declare that they have no competing interests.

## Funding

This study was funded by Capital's Funds for Health Improvement and Research (2020–2‐2072), Beijing Natural Science Foundation (L202005), Beijing Jishuitan Hospital “Medical and Engineering Intersection Project” (#YGQ‐201907), Beijing Jishuitan Hospital “Nova Program” (#XKXX201808), and Beijing Hospital Authority Youth Program (#QML20190403).

## Authors' Contributions

Da He and Wei He carried out the studies and drafted the manuscript. Bo Liu, Yajun Liu, Yuqing Sun, and Yonggang Xing performed the statistical analysis and participated in its design. Zhao Lang, Yumei Wang, Tengfei Ma, and Mingming Liu collected data. Wei Tian participated in acquisition, analysis, or interpretation of data and drafted the manuscript. All authors read and approved the final manuscript.
